# Protective effect of irbesartan against hepatic ischemia–reperfusion injury in rats: role of ERK, STAT3, and PPAR-γ inflammatory pathways in rats

**DOI:** 10.1007/s00210-024-03301-6

**Published:** 2024-08-21

**Authors:** Salma A. El-Marasy, Rasha E. Mostafa, Hoda B. Mabrok, Marwa S. Khattab, Sally A. El Awdan

**Affiliations:** 1 Department of Pharmacology, Medical Research and Clinical Studies Institute, National Research Centre, Giza, Egypt; 2 Nutrition and Food Science Department, Food Industries and Nutrition Research Institute, National Research Centre, Giza, Egypt; 3 Department of Pathology, Faculty of Veterinary Medicine, Cairo University, Giza, Egypt

**Keywords:** Irbesartan, Hepatic–ischemic reperfusion injury, ERK, STAT3, PPAR-γ inflammatory pathway, GW9662

## Abstract

This study aimed to elucidate the possible hepatocellular protective role of irbesartan during hepatic ischemia–reperfusion injury (HIRI) and the probable underlying mechanisms. Wistar rats were allocated into four groups: sham; HIRI (control); irbesartan (50 mg/kg) + HIRI; irbesartan (100 mg/kg) + HIRI; irbesartan + GW9662 (1 mg/kg, i.p.) + HIRI. Rats pretreated orally with irbesartan or vehicle for 14 days underwent 45-min hepatic ischemia followed by 60-min reperfusion. Irbesartan preconditioning diminished alanine transaminase (ALT) and aspartate transaminase (AST) serum levels, and reduced extracellular signal-regulated kinase (ERK) and signal transducer and activator of transcription 3 (STAT3). Irbesartan decreased proapoptotic BAX (bcl-2-like protein 4), increased anti-apoptotic B-cell lymphoma 2 (BCL2) hepatic content, and thereby reduced BAX/BCL2 ratio. Moreover, irbesartan preconditioning reduced autophagy-related proteins Beclin1 and LC3 II, and elevated p62 (protein responsible for autophagosome degradation). It elevated proliferator-activated receptor γ (PPAR-γ), and reduced tumor necrosis factor-alpha (TNF-α) and interleukin-6 (IL-6) hepatic gene expression. Also, hepatic protein expressions of nuclear factor kappa-B p65 (NF-κB p65) and caspase-3 were lessoned by irbesartan pretreatment in HIRI rats. However, GW9662 abrogated irbesartan's effect on HIRI. The protective effect of irbesartan on HIRI may be mediated by alleviation of ERK, STAT3, and PPAR-γ inflammatory pathways, exerting anti-apoptotic and anti-autophagic effects in HIRI in rats.

## Introduction

Hepatic ischemia–reperfusion injury (HIRI) takes place in the liver as a result of a variety of clinical and surgical conditions, including hemorrhagic shock, liver resection, liver transplantation, and hepatic trauma. HIRI may cause liver damage or loss of function, posing a significant treatment challenge (Scozzi and Gelman 2023).

A variety of factors might cause pathogenesis of HIRI, involving inflammatory cytokines, hepatocellular Ca^2+^ overload, oxidative stress, Kupffer cell activation, hepatic microcirculation impairment, and apoptosis (Choi and Lim 2023). During HIRI, liver cells, neutrophils, fat-storing cells, Kupffer cells, and hepatic sinusoidal endothelial cells interact (Nakatake et al. 2022). These activated cells release a huge amount of proinflammatory cytokines, which can contribute to additional inflammatory responses and cell death (Choi and Lim 2023). Reactive oxygen species damage hepatocytes and endothelial cells, activating Kupffer cells, and producing inflammatory mediators such as tumor necrosis factor-α (TNF-α) and interleukin (IL)-1β (Alaaeldin et al. 2023).

Extracellular signal-regulated kinase (ERK), a member of the mitogen-activated protein kinase family, exerts cellular effects through influencing the expression of several cytosolic proteins and nuclear transcription factors (Rezq et al. 2021). Signal transducer and activator of transcription 3 (STAT3) is a transcription factor that stimulates inflammatory pathways in rats with HIRI (Mahmoud et al. 2019). Activation of both ERK and STAT3 plays a vital role in HIRI (Xiong et al. 2018; Li et al. 2022).

Apoptosis is a sort of programmed cell death that occurs after HIRI for the first few hours following reperfusion and is one of the key death pathways of hepatocytes (Valdes et al. 2023). Autophagy, a kind of type 2 cell death that is crucial in hepatic damage, is distinguished from apoptosis by the existence of autophagosomes that are used for self-degradation in dying cells (Tang et al. 2023). As PPAR-γ is a negative macrophage regulator, it inhibits the generation of proinflammatory cytokines in HIRI in rats. Targeting PPAR-γ activation is a therapeutic concept to combat HIRI (Ding et al. 2021; Wu et al. 2021).

Irbesartan, a synthetic, nonpeptide antagonist of angiotensin II (Ang II), is primarily utilized as an angiotensin type 1 (AT1) receptor blocker for hypertension treatment (Darwish et al. 2021). Ang II activates AT1 receptors, triggering inflammation oxidative stress and apoptosis; thus, irbesartan combats those effects and exerts a hepatoprotective effect (Ertunc et al. 2024). Irbesartan has been proposed as a PPAR-γ ligand as well as its involvement in AT1 receptor blockade (Vignier et al. 2014). As PPAR-γ activation alleviates HIRI in rats (Morsy et al. 2022), irbesartan might be of value in the protection and alleviation of HIRI. Moreover, although irbesartan alleviated hepatic disorders in fructose-induced hepatic syndrome (Ibrahim et al. 2014), its effect on HIRI has not been studied yet.

As a result, various parameters were examined to investigate the probable protective impact of irbesartan in HIRI in rats. These parameters include serum alanine transaminase (ALT) and aspartate transaminase (AST) activities to investigate hepatocellular damage; hepatic contents of ERK, STAT3, and apoptotic signaling markers; and autophagic biomarkers, in addition to measuring mRNA expression of PPAR-γ and inflammatory cytokines. Histopathological and immunohistochemical examinations were conducted.

## Materials and methods

### Animals

Wistar rats weighing between 230 and 250 g were acquired from the National Research Centre's animal house in Egypt. The animals were kept in typical laboratory settings, with a 12-h light/dark cycle and a constant temperature of 25 ± 1 °C. During the week preceding the start of the experiment, rats were given regular laboratory pellets and water.

### Drugs and chemicals

Irbesartan was purchased from Sanofi Aventis Co. (Cairo, Egypt) and GW9962 from Sigma-Aldrich Co., USA. Analytical grade solvents and other chemicals were used in this study.

### Experimental design

Thirty rats were separated into four groups (six each) in this way: group 1—sham group that underwent midline laparotomy only; group 2—ischemia–reperfusion (I/R) group; groups 3 and 4—HIRI rats pretreated with irbesartan 50 and 100 mg/kg, respectively; group 5—HIRI rats pretreated with irbesartan and GW9662 (1 mg/kg; i.p.). Rats were orally pre-administered vehicle or irbesartan for 14 days, before HIRI. Doses of irbesartan and GW9662 were selected relevant to previous studies of Ilhan et al. (2022) and Mansour et al. (2022), respectively.

### Surgical procedure

All surgical procedures were performed under complete aseptic conditions, and intraperitoneal injections of ketamine 100 mg/kg and xylazine 10 mg/kg were administered anesthetic (Savvanis et al. 2014). Rats were exposed to midline abdominal incision, liver lobes and the portal triad identified, and rats were subjected to 45 min of hepatic ischemia by clamping the portal triad with a micro-vascular clamp after the bifurcation of the right lobe, interrupting the portal triad flow to the left and median lobes, inducing ischemia for 45 min, and after that the clamp removed to allow 60 min of reperfusion (Gheitasi et al. 2023). Blood samples were taken from the abdominal aorta and biopsies were extracted from the ischemic hepatic lobes at the end of the reperfusion period.

### Serum collection for analysis

Under sodium pentobarbital anesthesia, blood was drawn from the retro-orbital venous plexus and centrifuged (700 × *g*, 4°C, 20 min) to extract serum.

### Liver tissue extracts

Ten percent phosphate buffered saline was used to homogenize the liver. The first part was homogenized and centrifuged at 15,000 × *g* for 10 min. The supernatant was collected and stored at − 80°C to evaluate oxidative stress indicators and inflammatory cytokines. The second part was centrifuged at 5000 × *g* for 5 min and stored at − 80°C to measure the remaining parameters.

### Hepatic biochemical parameters in serum

Serum ALT and AST were determined by using colorimetric assay kits provided by Biomatik, Canada (catalogues EKU02211, EKE62019 respectively) in accordance with the manufacturer's instructions.

### Hepatic biochemical parameters in liver homogenate

#### ERK and STAT3

Based on the manufacturers' procedures, rat-specific enzyme-linked immunosorbent assay (ELISA) kits were used to determine the ERK (Abcam, Boston, USA) and STAT3 (MyBioSource, San Diego, USA) contents in liver homogenates.

### Determination of apoptotic markers

ELISA kits for apoptotic markers were employed to measure Bax (Cloud-Clone Corp., Katy, TX 77494, USA; catalog: SEA778Ra) and BCL2 (Cloud-Clone; catalog: SEB343Ra) hepatic content.

### Determination of autophagic markers

ELISA kits were used to assess Beclin II (Novus Biologicals, Littleton, CO; catalog number NBP2-69,960), LC3 II (Cell Biolabs Inc., San Diego, CA; catalog number CBA-5116), and P62 (Sunlong Biotech Co., China; catalog number SL1363Ra) hepatic content.

### mRNA expression of PPAR-γ, TNF-α, and IL-6

PurLink RNA-Mini-Kit (Ambion Life Technologies) was used to extract total RNA from frozen liver tissue in accordance with the manufacturer's protocol. Three micrograms of total RNA was used to synthesize cDNA with RevertAid first-strand cDNA synthesized kit (Thermo-Fisher Invitrogen) in accordance with the manufacturer's protocol. RT-PCR was performed with EvaGreen-qPCR-mastermix (Hot-FIREPol; Solis BioDyne) using a Rotor-Gene MDx instrument. Primer pair sequences used for proliferator-activated receptor (PPAR-γ), tumor necrosis factor-α (TNF-α), interleukin-6 (IL-6), and glyceraldehyde-3-phosphate dehydrogenase (GAPDH) are presented in Table 1. Target gene expression was normalized with GAPDH gene expression (housekeeping gene) and delta-delta-CT method (Livak and Schmittgen, 2001) was used to calculate the relative fold gene expression.

**Table 1:** Primer pair sequences used for real-time PCR amplifications

Target genes	Sequences	Annealing temperature (°C)	Reference
PPAR-γ	FW (5′-TTATAGCTGTCATTATTCTCAGTGGA-3′) RW(5′-CGGGTGGTTCAGCTTCAG-3′)	60	(Tomaz et al. 2016)
TNF-α	FW (5′-ACTGAACTTCGGGGTGATTG-3′) RW (5′-GCTTGGTGGTTTGCTACGAC-3′)	60	(Khan et al. 2013)
IL-6	FW (5′-TGA TGG ATG CTT CCA AAC TG-3′) RW (5′-GAG CAT TGG AAG TTG GGG TA-3′)	60	(Khan et al. 2013)
GAPDH	FW (5′-GTATTGGGCGCCTGGTCACC-3′) RW (5′-CGCTCCTGGAAGATGGTGATGG-3′)	60	(Khan et al. 2013)

### Histopathology

Liver specimens were processed using the paraffin embedding procedure after being preserved in 10% neutral buffered formalin. Following tissue sectioning into 4-µm-thick slices, hematoxylin and eosin stain was applied. For the examination, a light microscope with a digital camera was utilized.

Three histological lesions in HIRI were scored according to the methods described by Suzuki et al. (1993), which included venous congestion, vacuolization, and necrosis all ranked in a scale from 0 to 4.

### Immunohistochemistry

Following deparaffinization, rehydration, and antigen retrieval using citrate buffer (pH 6), primary antibodies against NF-κB P65 (sc-8008; Santa Cruz Biotechnology, Inc., Santa Cruz, CA, USA) were employed. Following the manufacturer's instructions, a secondary horseradish peroxidase (HRP)–labeled antibody was applied (Universal PolyHRP DAB kit for mouse and rabbit; Genemed, Sakura, Torrance, CA, USA). Similarly, the caspase-3 immunohistochemistry was performed using caspase-3 primary antibodies (Novus Biologicals, Centennial, CO, USA). Hematoxylin was employed as the counterstain and diaminobenzidine as the substrate. Three images/samples were used to calculate the percentage area of positively stained tissue using ImageJ software.

### Statistical analysis

Data were expressed as mean ± standard error of different parameters between treated groups. Tukey's post hoc test was performed following one-way analysis of variance (ANOVA). GraphPad Prism 7 software was used for statistical analysis. The values were considered significant when *p* < 0.05.

## Results

### AST and ALT serum levels in HIRI in rats treated with irbesartan

In Table 2 I/R caused a substantial deterioration of liver function as manifested by an elevation in serum AST and ALT activities to 383 and 370%, respectively, as compared to the sham-operated group.

**Table 2:** Effect of irbesartan on AST and ALT serum levels in HIRI in rats

Parameters Groups	AST (ng/ml)	ALT (ng/ml)
Sham	3.27^bc^ ± 0.18	7.80^bc^ ± 0.72
I/R	12.53^a^ ± 0.64	28.87^a^ ± 2.63
I/R + irbesartan (50 mg/kg)	6.57^abc^ ± 0.37	17.30^abc^ ± 0.4
I/R + irbesartan (100 mg/kg)	3.57^bc^ ± 0.21	11.90^bc^ ± 0.77
I/R + GW9662 + irbesartan (50 mg/kg)	11.40^a^ ± 0.38	24.80^a^ ± 0.58

Data is presented as means ± standard error of means (*n* = 6)

*AST* aspartate transaminase, *ALT* alanine transaminase

^a^Versus sham-operated group

^b^Versus I/R group

^c^Versus I/R + GW9662 + irbesartan (50 mg/kg) group at *p* ≤ 0.05

Irbesartan treatment (50 mg/kg) significantly decreased AST and ALT activities to reach 52 and 60%, respectively, while irbesartan treatment (100 mg/kg) significantly decreased AST and ALT activities to reach 29 and 41%, respectively, as compared to I/R control group.

Combining the PPAR-γ blocker, GW9662 with irbesartan (50 mg/kg) resulted in a reduction in the irbesartan effect as manifested by AST and ALT activities decreasing to only 86 and 70% of the I/R control group.

### ERK and STAT3 contents in HIRI in rats treated with irbesartan

As observed in Table 3 I/R caused an extensive elevation of liver tissue ERK and STAT3 contents to 280 and 276%, respectively, as compared to the sham-operated group.

**Table 3:** Effect of irbesartan on ERK and STAT3 contents in HIRI in rats

Parameters Groups	ERK (μg/mg protein)	STAT3 (ng/mg protein)
Sham	1.13^ab^ ± 0.11	0.70^ab^ ± 0.05
I/R	3.16^a^ ± 0.09	1.93^a^ ± 0.09
I/R + irbesartan (50 mg/kg)	2.08^abc^ ± 0.08	1.32^abc^ ± 0.02
I/R + irbesartan (100 mg/kg)	1.35^bc^ ± 0.06	0.81^bc^ ± 0.03
I/R + GW9662 + irbesartan (50 mg/kg)	2.88^ab^ ± 0.10	1.80^a^ ± 0.07

Data is presented as means ± standard error of means (*n* = 6)

*ERK* extracellular signal-regulated kinase, *STAT3* signal transducer and activator of transcription 3

^a^Versus sham-operated group

^b^Versus I/R group

^c^Versus I/R + GW9662 + irbesartan (50 mg/kg) at *p* ≤ 0.05

Irbesartan treatment (50 mg/kg) significantly decreased the liver tissue ERK and STAT3 contents to reach 43 and 68%, respectively, while irbesartan treatment (100 mg/kg) significantly decreased liver tissue ERK and STAT3 levels to reach 43 and 42%, respectively, as compared to the I/R control group.

Administration of GW9662 with irbesartan (50 mg/kg) resulted in a decline in irbesartan's effect as manifested by a decrease in ERK content to only 91% of the I/R control group while there was no change in STAT3 content.

### Apoptotic parameters in HIRI in rats treated with irbesartan

**Fig. 1:**
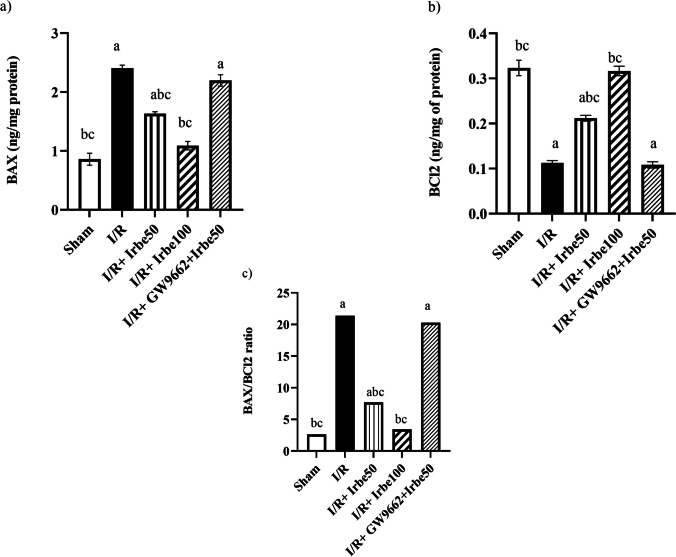
Effect of irbesartan on** a** BAX,** b** BCL2, and** c** BAX/BCL2 ratio in HIRI in rats. Data is presented as means ± standard error of means (*n* = 6). ^a^ vs sham-operated group, ^b^ vs I/R group, and.^c^ vs I/R + GW9662 + irbesartan 50 group at *p* ≤ 0.05

As distinguished in Fig. 1, I/R caused an extensive rise of liver tissue BAX to reach 279% accompanied by a significant decline in BCL2 levels to reach 34% as compared to the sham-operated group. The net result is a significant increase in the BAX/ BCL2 ratio.

Irbesartan treatment (50 and 100 mg/kg) significantly decreased the hepatic BAX content to reach 68 and 45%, respectively, while it increased hepatic BCL2 content to reach 191 and 291%, respectively, as compared to the I/R control group. No change was detected in the administration of GW9662 to irbesartan treatment. Irbesartan treatment consequently decreased the elevated BAX/BCL2 ratio dose dependently.

### Autophagic parameters in HIRI in rats treated with irbesartan

**Fig. 2:**
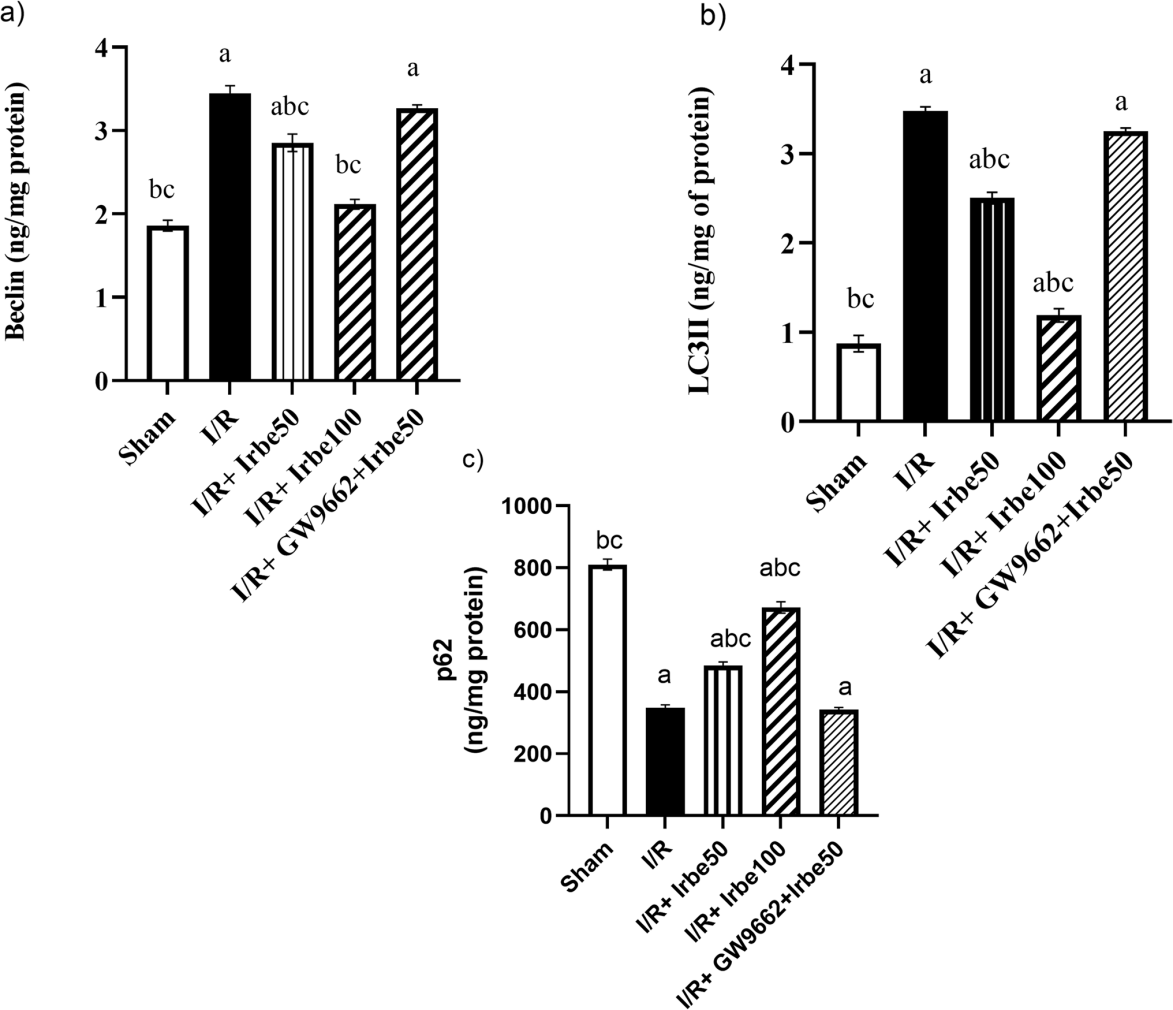
Effect of irbesartan on** a** Beclin,** b** LC3 II, and** c** P62 contents in HIRI in rats. Data is presented as means ± standard error of means (*n* = 6). ^a^ vs sham-operated group, ^b^ vs I/R group, and.^c^ vs I/R + GW9662 + irbesartan 50 group at *p* ≤ 0.05

I/R caused a significant elevation of Beclin and LC3 II hepatic contents to reach 185 and 400%, respectively, accompanied by a significant decline in P62 levels to reach 43% as compared to the sham-operated group in Fig. 2.

Irbesartan treatment (50 mg/kg) significantly decreased the raised hepatic Beclin and LC3 II contents to reach 83 and 72%, respectively, while it increased P62 hepatic content to reach 137% as compared to the I/R control group.

In comparison, irbesartan treatment (100 mg/kg) significantly decreased the raised Beclin and LC3 II hepatic contents to reach 74 and 48%, respectively, while it increased P62 hepatic content to reach 191% as compared to the I/R control group.

Cotreatment of GW9662 with irbesartan (50 mg/kg) implicated no change in Beclin, LC3 II, and P62 levels compared to the I/R control group.

### qRT-PCR mRNA gene expression of PPAR-γ, TNF-α, and IL-6 in HIRI in rats treated with irbesartan

The gene expression of PPAR-γ, TNF-α, and IL-6 in liver was measured. I/R significantly inhibited the mRNA expression of PPAR-γ (*p* ≤ 0.001) compared to the sham-operated group. In comparison, irbesartan treatments (50 and 100 mg/kg) reversed this inhibition and significantly up-regulated (*p* ≤ 0.001) the mRNA expression of PPAR-γ with 2.0- and 2.5-fold change, respectively. An elevation of PPAR-γ expression was observed after treatment with GW9662 (PPAR-γ blocker) and irbesartan (50 mg/kg) with a 1.7-fold change (*p* ≤ 0.001) compared to the I/R control group.

TNF-α and IL-6 gene expressions were markedly elevated in the I/R control group compared to the sham-operated group. The liver mRNA expression of TNF-α and IL-6 was significantly down-regulated (*p* ≤ 0.001) in all treatment groups. Irbesartan treatments (50 and 100 mg/kg) and irbesartan (50 mg/kg) + PPAR-γ blocker decreased the level of TNF-α by 2.24-, 3.04-, and 1.91-fold, respectively, compared to the I/R control group.

Likewise, IL-6 levels were decreased by 3.01-, 4.06-, and 2.16-fold, respectively, compared to the I/R control group. There is no significant difference between irbesartan treatments (50 mg/kg) and irbesartan (50 mg/kg) + PPAR-γ blocker (Table 4).

**Table 4:** The qRT-PCR mRNA gene expression of PPAR-γ, TNF-α, and IL-6 in HIRI in rats

**Parameters** **Groups**	**mRNA relative gene expression (fold change)**		
	**PPAR-γ**	**TNF-α**	**IL-6**
Sham	1.002 ± 0.042	0.254 ± 0.010	0.177 ± 0.007
I/R	0.329^a^ ± 0.011	1.002^a^ ± 0.046	1.005^a^ ± 0.074
I/R + irbesartan (50 mg/kg)	0.663^ab^ ± 0.007	0.446^ab^ ± 0.014	0.334^b^ ± 0.014
I/R + irbesartan (100 mg/kg)	0.832^abc^ ± 0.019	0.329^bc^ ± 0.005	0.248^bc^ ± 0.026
I/R + GW9662 + irbesartan (50 mg/kg)	0.563^ab^ ± 0.012	0.523^ab^ ± 0.010	0.465^ab^ ± 0.019

The mRNA expressions of PPAR-γ, TNF-α, and IL-6 are normalized with the housekeeping gene (GAPDH), and values are represented as ± SEM (*n* = 6)

^a^Versus sham-operated group

^b^Versus I/R group

^c^Versus I/R + GW9662 + irbesartan (50 mg/kg) at *p* ≤ 0.05

### Effect of irbesartan on histopathological changes

**Fig. 3:**
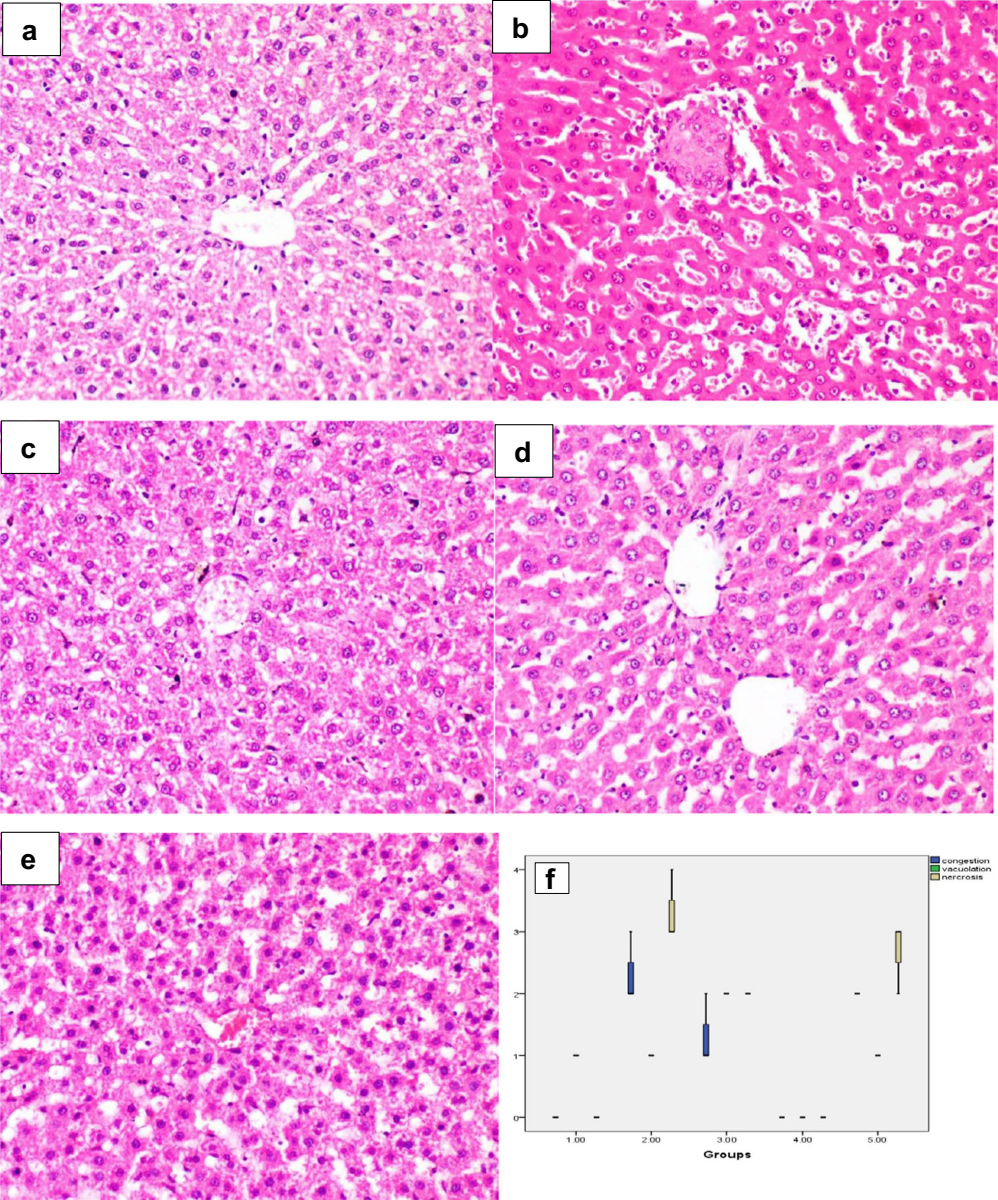
Histopathology of liver in different groups. **a** Mild histopathological alteration in the liver of the sham group; **b** sinusoidal dilatation with leukocytosis, apoptotic bodies, and fibrin thrombus in the central vein in I/R group; **c** moderate histopathological alteration in the I/R + irbesartan (50 mg/kg) group; **d** mild histopathological alteration in the I/R + irbesartan (100 mg/kg) group; **e** centrilobular hepatocyte necrosis in the I/R + irbesartan (50 mg/kg) + GW9662 (1 mg/kg) group. **f** Boxplot of lesion scores of venous congestion, vacuolation, and necrosis in different groups. The boxes are the interquartile range (IQR). The medians are the thick middle lines. The maximum and minimum values are represented by the thin horizontal lines at the top and bottom. Hematoxylin and eosin stain × 200

Microscopy of the liver revealed mild histological alteration in the sham group (Fig. 3a); however, in the I/R group, there were severe sinusoidal dilatations with leukocytosis, apoptotic bodies, centrilobular necrosis, occasional fibrin thrombus in the central vein, and hemorrhage (Fig. 3b). In the I/R + irbesartan (50 mg/kg) group, the lesions observed were partially regressed compared to the I/R group (Fig. 3c). Also, in the I/R + irbesartan (100 mg/kg) group, the lesions were greatly regressed compared to previous groups (Fig. 3d). In the I/R + irbesartan (50 mg/kg) + GW9662 (1 mg/kg) group, the lesions were comparable to those observed in the I/R group (Fig. 3e). Lesion scoring in different groups indicated a significant increase in venous congestion, vacuolation, and necrosis in the I/R group and I/R + irbesartan (50 mg/kg) + GW9662 (1 mg/kg) group, which however was decreased in treated groups especially in high-dose I/R + irbesartan (100 mg/kg) group (Fig. 3f).

### Effect of irbesartan on immunohistochemistry examination

**Fig. 4:**
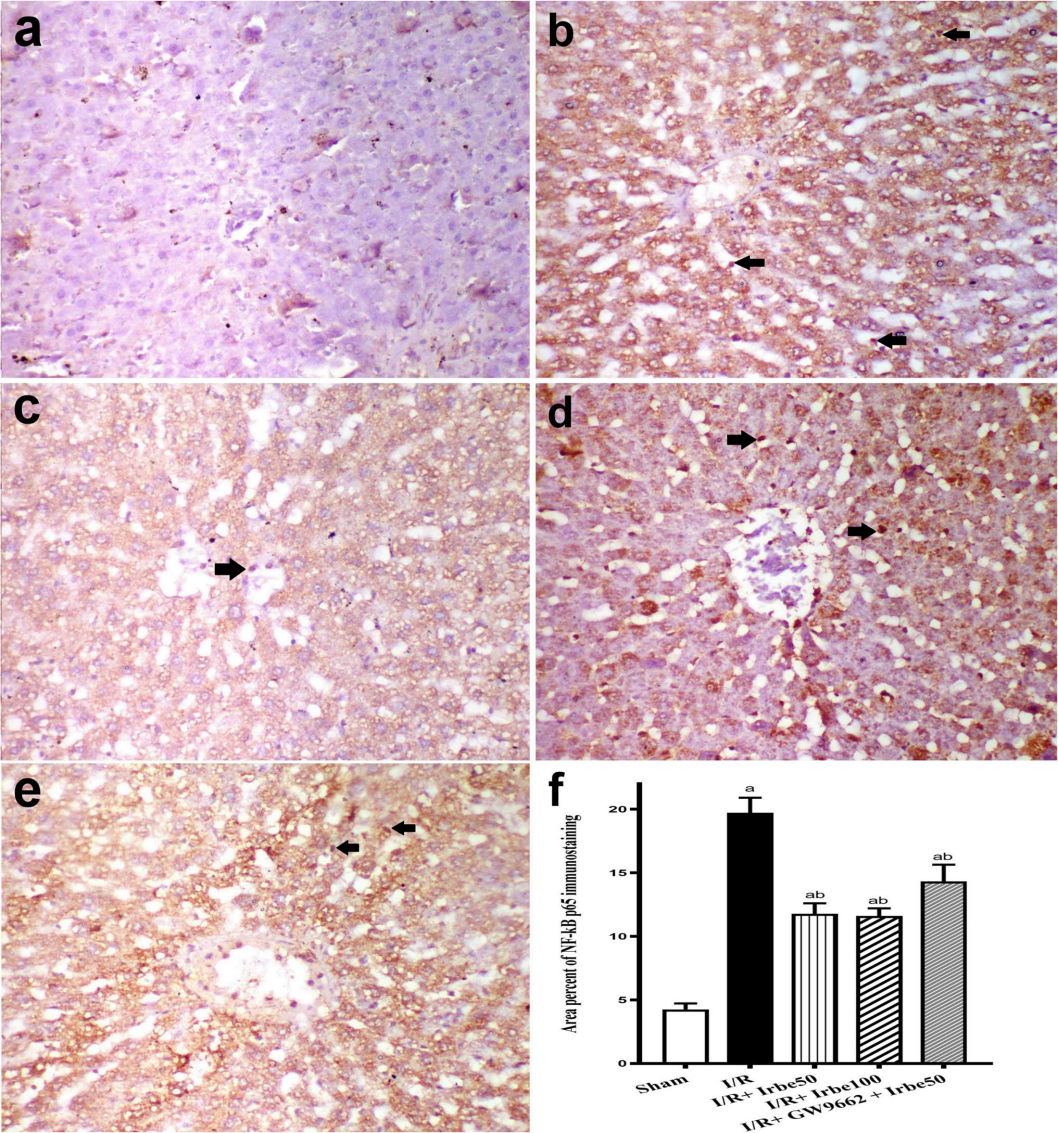
Immunohistochemistry of NF-κB p65 in liver of different groups. **a** Weak NF-κB expression in sham group, **b** severe NF-κB p65 expression in hepatocyte cytosol and nuclei of Kupffer cells (arrows) in I/R group, **c** moderate NF-κB p65 expression in hepatocyte cytosol and Kupffer cell nuclei (arrow) in the I/R + irbesartan (50 mg/kg) group, **d** mild NF-κB p65 expression in hepatocyte cytosol and nuclei (arrows) in the I/R + irbesartan (100 mg/kg) group, **e** moderate expression in hepatocyte cytosol and Kupffer cell nuclei (arrows) in the I/R + irbesartan (50 mg/kg) + GW9662 (1 mg/kg) group, **f** mean values of area percent of NF-κB p65 in liver of different groups. Columns bearing different lowercase letters are considered significant at *p* < 0.05. Immunoperoxidase and hematoxylin counterstain × 200

Weak NF-κB p65 expression was observed in the sham group (Fig. 4a). Severe NF-κB expression was recorded in the I/R group (Fig. 4b). Moderate NF-κB p65 protein expression was observed in the I/R + irbesartan (50 mg/kg) group (Fig. 4c). Mild NF-κB p65 expression was recorded in the I/R + irbesartan (100 mg/kg) group (Fig. 4d). Moderate expression was noticed in the I/R + irbesartan (50 mg/kg) + GW9662 (1 mg/kg) group (Fig. 4e). The area percent of NF-κB p65 expression in different groups is represented in Fig. 4f.

**Fig. 5:**
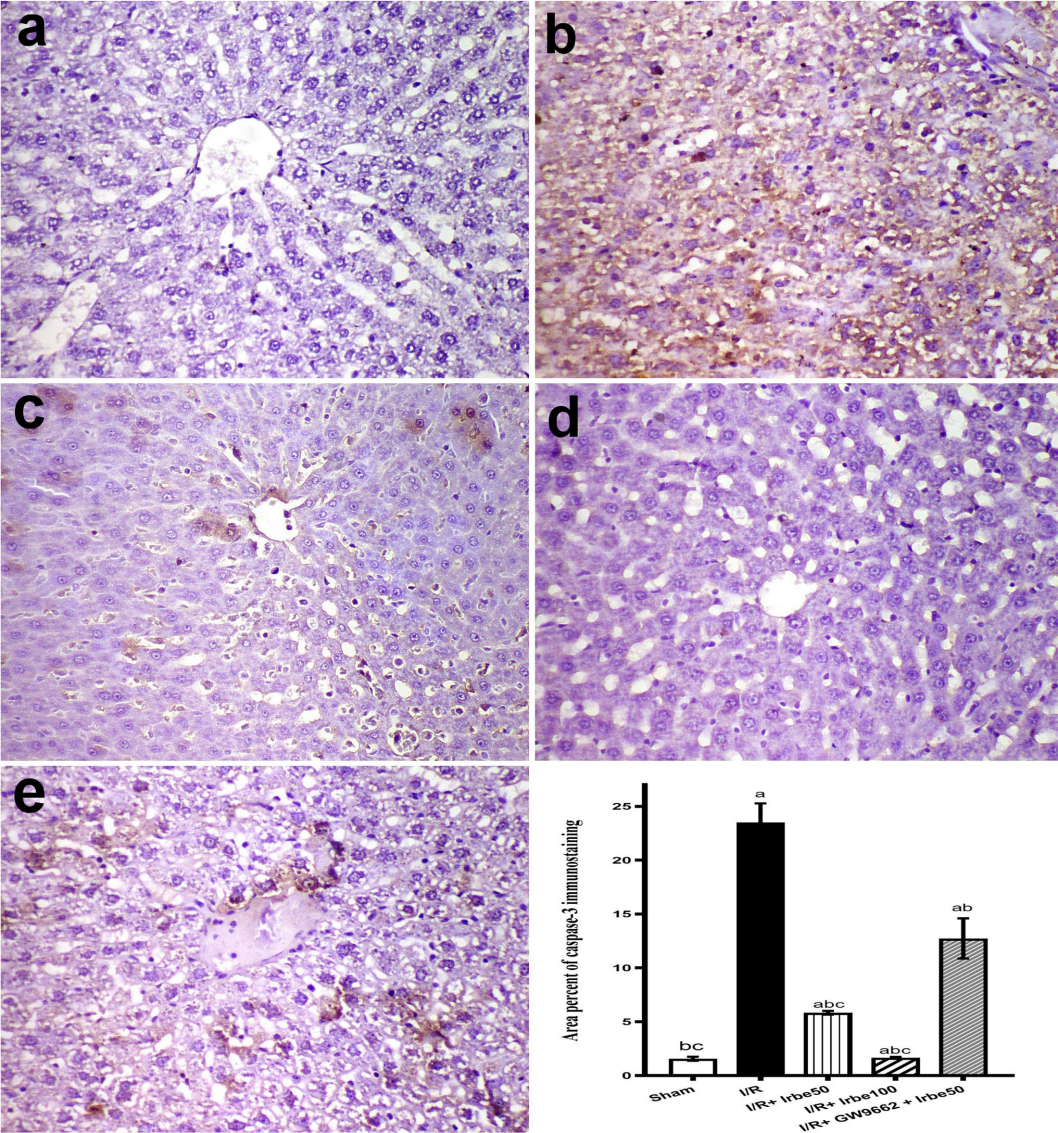
immunohistochemistry of caspase-3 in liver of different groups. **a** Weak caspase expression in sham group, **b** severe caspase expression in I/R group, **c** moderate caspase expression in the I/R + irbesartan (50 mg/kg) group, **d** mild caspase expression in the I/R + irbesartan (100 mg/kg) group, **e** moderate expression in the I/R + irbesartan (50 mg/kg) + GW9662 (1 mg/kg) group. **f** Mean values of area percent of caspase-3 in liver of different groups. Columns bearing different lowercase letters are considered significant at *p* < 0.05. Immunoperoxidase and hematoxylin counterstain × 200

Caspase-3 was weakly expressed in the sham group (Fig. 5a). Severe caspase expression was observed in the I/R group (Fig. 5b). Moderate caspase expression in the I/R + irbesartan (50 mg/kg) group (Fig. 5c) and mild caspase expression in the I/R + irbesartan (100 mg/kg) group were recorded (Fig. 5d). Moderate expression was observed in the I/R + irbesartan (50 mg/kg) + GW9662 (1 mg/kg) group (Fig. 5e). The area percent of caspase expression in different groups is represented in Fig. 5f.

## Discussion

HIRI is a pathological condition caused by insufficient blood flow to the liver, after which perfusion is restored and reoxygenation occurs (Fang et al. 2023). Restoration of blood supply to ischemic liver tissue led to remarkable hepatocellular damage, elevated inflammatory response, hepatocyte apoptosis, and autophagy (Yao et al. 2023). In harmony with prior investigators, this study implies that 60 min of hepatic reperfusion preceded by 45 min of ischemia contributed to a marked elevation in hepatocellular enzymes ALT and AST. Moreover, HIRI elevated ERK, STAT-3, and PPAR-γ inflammatory pathways, evidenced by the elevation of ERK, STAT3 hepatic contents, reduction in PPAR-γ gene expression, elevation in NF-κB p65 protein expression, TNF-α as well as IL-6 gene expressions. Furthermore, herein, HIRI was accompanied by hepatocyte apoptosis witnessed by elevation of proapoptotic BAX hepatic content, reduction in anti-apoptotic BCL2 hepatic content, elevation in BAX/BCL2 ratio, and caspase-3 protein expression. It is worthy to state that HIRI potentiated autophagy, as it elevated Beclin and LCII and reduced p62. All those findings were supported by histopathological alterations depicted in HIRI in rats.

Irbesartan is a selective PPAR-γ agonist and Ang II receptor blocker that has been reported to guard against drug-induced nephrotoxicity, cardiotoxicity, and hepatotoxicity (Tepebasi et al. 2023; Ertunc et al. 2024; Pastaci Ozsobaci et al. 2024). To the authors' knowledge, this research discloses novel evidence on the protective effect of irbesartan (50, 100 mg/kg) on HIRI in rats. The role of irbesartan on ERK, STAT3, and PPAR-γ inflammatory pathways, apoptosis, autophagy, and histopathological changes in HIRI in rats was assessed.

Rat HIRI is a well-known model that is verified by elevation in liver enzymes and alterations in histopathological examination (Mahmoud et al. 2023). In this study, irbesartan ameliorated hepatic dysfunction resulting from IR injury as it restored ALT and AST hepatic enzymes and attenuated hepatic histopathological changes, revealing its hepatoprotective effect against HIRI. In accordance, irbesartan protected against hepatocellular damage induced by acetaminophen in mice (Helal and Samra 2020).

In this study, irbesartan-treated IR rats reduced hepatic ERK content, revealing that its hepatoprotective effect in I/R injury was attributed to modulation of ERK pathway. ERK belongs to the family of mitogen-activated protein kinases (MAPK) signaling mediators. It is a pivotal protein mediator that is involved in cell differentiation, proliferation, and apoptosis. Several studies have reported the inhibition of ERK pathway in protection against HIRI in rats (Zhang et al. 2023). Also, deactivation of ERK pathway by irbesartan mediated its cardioprotective effect against myocardial I/R injury and its gastroprotective properties against indomethacin-induced gastric damage in rats (Shahin et al. 2018; Ren et al. 2019).

In the current work, irbesartan reduced STAT3 hepatic content in HIRI in rats. After activation with cytokines or growth factors and attaching to a cell-surface receptor, STAT factors are rapidly tyrosine phosphorylated, dimerized, and transported to the nucleus, where they attach to specific hormone-sensitive regions of the target genes, triggering an inflammatory cascade (Zhang et al. 2019).

Moreover, irbesartan decreased the hepatic protein expression of NF-κB p65 and reduced the hepatic gene expressions of TNF-α and IL-6 in IRI in rats. NF-κB is a crucial transcription factor that mediates inflammatory responses and provokes the secretion of TNF-α and IL-6 cytokines (Mohammed et al. 2020). Activation of STAT3 is correlated with the NF-κB inflammatory pathway in HIRI in rats (Kamel et al. 2020). Therefore, it can be deduced that irbesartan exerted its hepatoprotective effect via inhibition of STAT-3/NF-κB-dependent inflammatory pathway in IRI in rats.

It is noteworthy to mention that activation of ERK pathway promotes inflammation in HIRI in rats via activation of the NF-κB pathway (Jiang et al. 2022). Thus, irbesartan's anti-inflammatory effect might also be moderated by inhibition of ERK/NF-κB pathway in HIRI in rats. Previous studies stated that AT1 receptor blocker inhibited STAT3 inflammatory pathway in hepatic liver fibrosis (Fouad et al. 2022). In the same line, mounting studies demonstrated a hepatoprotective effect of irbesartan via suppression of acetaminophen and doxorubicin-induced hepatocyte inflammation in rats (Kabel et al. 2018; Helal and Samra 2020).

Herein, irbesartan-treated IRI elevated PPAR-γ hepatic gene expression, whereas coadministration with GW9662 combated this elevation. This implies that irbesartan moderated its hepatoprotective effect through activation of PPAR-γ pathway and thereby inhibiting inflammation.

Consistently, irbesartan attenuated liver steatosis in diabetic db/db mice through stimulation of PPAR-γ and thereby inhibiting inflammatory cascade (Zhong et al. 2017). Moreover, irbesartan protected against testicular damage induced by cyclophosphamide by activating PPAR-γ pathway (Abu-Risha et al. 2022).

The present work demonstrated that irbesartan reduced proapoptotic protein BAX, elevated anti-apoptotic BCL2 hepatic contents, reduced BAX/BCL2 ratio which indicates the degree of apoptosis, and reduced caspase-3 hepatic protein expression, the final product of apoptosis. BCL2 combats apoptosis by hindering BAX activity and thereby hampering caspase activation. Those findings confer that irbesartan's anti-apoptotic effect mediated its hepatoprotective effect in HIRI in rats. Consistently, irbesartan repressed apoptosis in hepatotoxicity induced by doxorubicin and gastric injury induced by ethanol in rats (Kabel et al. 2018; Abu-Risha et al. 2022).

Beclin1, LC3 II, and p62 are markers of autophagy (Jovanovic et al. 2022). Irbesartan in this work reduced Beclin1, proautophagy protein, and LC3 II, and elevated p62 hepatic contents, revealing that irbesartan combated hepatocellular autophagy throughout HIRI. A previous study verified that irbesartan ameliorated ovarian IR via halting apoptosis (Hortu et al. 2020).

BCL2 is an intermediary protein between apoptosis and autophagy, which is coupled by Beclin1/BCL2 complex. The elevated BCL2 binds with Beclin1 and reduces the conversion of LC3 I to LC3 II. This conversion is essential for formation of autophagosomes (Mao et al. 2023). Reduced Beclin1 restricts its ability to bind to the ubiquitin-binding protein p62, which carries LC3 II and thereby inhibits autophagosome degradation and combats apoptosis (Li et al. 2023). This notion reveals that irbesartan's anti-apoptotic effect moderated its inhibitory action on autophagy in HIRI.

Xiang et al. (2020) reported that PPAR-γ activation directly upregulates BCL2 and reduces BAX; therefore, it can be deduced that irbesartan combated apoptosis and autophagy via activating PPAR-γ pathway. It is worthy to mention that ERK/NF-κB activation mediates apoptosis and autophagy (Yu et al. 2019; Ji et al. 2020). Irbesartan's action on apoptosis and autophagy was moderated by abating ERK/NF-κB activation.

## Conclusion

In conclusion, irbesartan's hepatoprotective effect was intervened by attenuation of ERK, STAT3, and PPAR-γ inflammatory pathways, apoptosis, and autophagy in HIRI in rats. Further research is warranted to elucidate other pathways and molecular mechanisms underlying the hepatoprotective effect of irbesartan in HIRI.

## Data Availability

Available upon request.
